# Emotions, Perceived Stressors, and Coping Strategies Among Nursing Staff in Saudi Arabia During the COVID-19 Pandemic

**DOI:** 10.7759/cureus.48284

**Published:** 2023-11-04

**Authors:** Lamees S Bakhsh, Abeer AlHazmi, Alla BaMohammed, Eiman Binishaq, Ghadah Abdullah, Razaz Bajal, Ibrahim Al Ramamneh

**Affiliations:** 1 Department of Nursing, King Abdulaziz University Hospital, Jeddah, SAU; 2 Department of Nursing, King Abdulaziz University Faculty of Medicine, Jeddah, SAU

**Keywords:** saudi, stressors, coping, nurses, first wave, covid-19

## Abstract

Objective

The COVID-19 pandemic resulted in heightened stress for nurses and other healthcare workers, particularly during the initial phase of the crisis. Despite the adoption of various coping strategies, psychological distress persisted, affecting nurses' well-being and jeopardizing the overall resilience of the healthcare system. This study assessed the emotional response, perceived stressors, and coping strategies among nurses’ staff who worked during the first wave of COVID-19 pandemic.

Method

A cross-sectional study was carried out among nurses who worked during the initial phase of the COVID-19 crisis (June - August 2020), at a tertiary care center in Western Saudi Arabia. The questionnaire explored five main sections. The first section (15 items) assessed emotions experienced during the initial wave of COVID-19, capturing both positive and negative sentiments, such as “joy” or “fear.” The second section (20 items) examined the presence of stressors, like “lack of protective equipment” or “fear of infection.” The third section (14 items) evaluated the perceived effectiveness of certain stress-reducing factors, including “peer support” or “training.” In the fourth section (13 items), participants rated their usage frequency of various coping strategies, such as “meditation” or “seeking advice.” Lastly, the fifth section assessed the hypothetical impact of 10 incentives, like “financial bonuses” or “additional training,” in motivating nurses' involvement in future epidemic responses. The questionnaire was completed with demographic and professional data. A convenience sampling method was employed, and 315 nurses participated in the study. Descriptive statistics were carried out using SPSS version 24 for Windows (IBM Corp., Armonk, NY).

Result

The most commonly experienced emotion was a feeling of responsibility and ethical duty, reported by 97.5% of the participants, followed by nervousness and fear (83.8%), anger (73.3%), and stigma (70.2%). On the other hand, 86.7% were expecting a financial compensation. The most common stressors were related to the nurses’ own safety, or the safety of their families and colleagues, reported by 92.4-95.2% of the participants. The perceived uncontrollability of COVID-19 was also a significant stressor. The improvement of the health status of infected colleagues (98.1%) or patients (97.5%) were the most common factors associated with the reduction in nurses’ stress. Among the coping strategies, five were almost systematically deployed by the nurses (>95%), all consisting of cognitive and behavioral mechanisms to enhance own knowledge and safety and avoid being infected. The most crucial determinants for commitment in future pandemics are the availability of a cure or vaccine (93.3%), family support (91.4%), adequate personal protective equipment from the hospital (90.8%), and exemption from overtime (90.2%).

Conclusion

The first wave of COVID-19 exerted a tremendous psychological stress on nurses, due to concerns about safety, disease uncertainties, and social isolation. Analyzing these impacts offers insights for enhancing institutional and national crisis strategies, emphasizing staff safety and psychological well-being, especially for first responders like nurses. Policy implications include prioritizing mental health support and preparedness in future crisis plans. Additionally, ensuring continuous training and strategic workload management is crucial for maintaining frontline commitment.

## Introduction

At the end of 2019, the World Health Organization (WHO) announced a cluster of pneumonia cases of an unknown cause in Wuhan City, which rapidly became one of the deadliest and most expansive pandemics of all time. The causative agent was identified to be a coronavirus, named SARS-CoV-2, and the disease was termed COVID-19 [1]. The extent of damage caused by COVID-19 on human society was substantial, with inestimable direct and far-reaching effects on lives, health, economy, and overall well-being, notably psychological well-being [2,3]. Globally, mental health has been affected to a great extent, and it has been the most extensively studied subject during the pandemic COVID-19. The brutal onset of the pandemic has resulted in high levels of psychological distress and anxio-depressive disorders; not only concerning infected individuals but also the general population [4,5]. The amount of these consequences enlarged the health and economic disparities within the populations due to the higher burden incurred by vulnerable groups [6,7].

Among the subgroups who were particularly subjected to stress are the nurses, who have been a central part of the first responders. In Saudi Arabia and internationally, substantial levels of stress and burnout have been recorded among nurses and other healthcare workers (HCWs) during the first wave of the pandemic [8-12]. The emergency of the crisis combined with other uncontrollable stressor, such as the lack of information about the disease, the absence of effective preventive and therapeutic guidelines, and the heaviness of the restrictive measures, mixed with isolation and uncertainties, have intensified the job-related stress among HCWs [9,13,14]. The levels of psychological distress and suffering remained high despite the deployment of various coping strategies [15-17]. This resulted in insufficient resilience and a cumulative traumatic experience that impacted the HCWs' well-being and productivity; thus, endangering the resilience of the healthcare system [18,19].

In Saudi Arabia, prior to COVID-19, HCWs have confronted numerous epidemics over the past two decades, underscoring the importance of drawing lessons from past experiences to enhance future preparedness and response. One example of these past experiences was the outbreak of an unexplained hemorrhagic fever in the South-Western border of Saudi Arabia and Yemen, in 2000, which had a mortality rate of 14% [20,21]. More recently, in 2014, the outbreak of sub-acute respiratory syndrome (SARS), caused by the Middle East respiratory syndrome coronavirus (MERS-CoV), which originated from Saudi Arabia, resulted in 300 cases and 40 deaths [22,23]. Subsequent data showed that HCWs accounted for 21% of the SARS cases, which generated high levels of stress, emotional distress, and fear among all the exposed healthcare providers [24]. Further studies that explored the psychological impact of the SARS outbreak indicated that up to 78% of the HCWs, including nurses, had significant or high levels of stress and fear [25,26].

The defense mechanisms against stress have been an important topic of several previous studies [27]. In critical situations, individuals’ response to stress varies according to their level of resilience and their coping strategy [28]. Coping strategies refer to cognitive and behavioral mechanisms that help in reducing the mental pressure of a stressful situation. Nonetheless, the relationship between the level of stress, the emotional response, and the effectiveness of coping strategies may be paradoxical [29,30]. A study from Italy during COVID-19 showed that higher levels of stress among HCWs were associated with adverse coping behaviors, such as exposure to infected patients without optimal personal protective equipment (PPE). On the other hand, positive coping strategies, such as social support, problem-solving, and positive attitude, were deployed in HCWs who experienced lower levels of stress [27]. This demonstrates the need to explore the pattern of coping strategies and their relationship with stress and its emotional impact.

The present study assessed the emotional response, perceived stressors, and coping strategies among nursing staff who worked during the first wave of the COVID-19 pandemic. Such information offers important insights and implications that could guide interventions and policymaking, ensuring the resilience and well-being of these frontline professionals in subsequent health emergencies.

## Materials and methods

Design and setting

A descriptive cross-sectional study was carried out at King Abdulaziz University Hospital (KAUH), Jeddah, Saudi Arabia. KAUH is a tertiary hospital with a bed capacity of 1,152. The study was conducted during the period June-August 2020, corresponding to the peak of COVID-19 cases in the country. During this period, Saudi Arabia experienced significant fluctuations in its COVID-19 situation. Starting from an initial 392 cases in March 2020, the country witnessed a rapid escalation of the incident number of daily cases. However, by the end of June, a promising decline in daily infections emerged, decreasing from 4,757 cases on June 18 to 220 by November 28. Active cases peaked in mid-July at 63,026 but subsequently began to decrease. Critical cases reached their height at 2,295 on July 4, 2020. While the daily rise in infections touched its maximum, with an increase of 810 cases on July 3, it started decreasing from August. Despite these challenges, the healthcare response was commendable, as evidenced by a higher number of recoveries than new infections from May 12, 2020, onward [31].

Population

The study included nurses who were working at the hospital during the study period. All departments were included such as emergency department, isolation, general units, etc. We also included cross-trained staff, corresponding to staff being moved to different specialty units to cover eventual shortages and who required training. However, nursing students were excluded. 

Sampling

A total of 1,237 nurses were estimated to be eligible to participate in the study. The sample size was calculated to detect an unknown proportion (P=50%) corresponding to the percentage of a given emotional response, with 0.05 type 1 error and 80% statistical power. The target sample size was calculated at N=294. Convenience sampling was used to involve all eligible and consenting nurses, until reaching the target sample size.

Tool

The questionnaire originally adapted and utilized by Khalid et al. [25] for hospital HCWs during the MERS-CoV outbreak in 2014, consisted of five sections with a total of 72 questions. On average, respondents took about 20 minutes to complete it. The questionnaire's sections were the following.

The first section consisted of 15 Likert-type scale items that explored the intensity (from 0 = not at all, to 3 = very much) of 15 different emotions experienced while working during the first wave of COVID-19. Emotions were positive or negative, depending on the item. The internal consistency coefficients were calculated at 0.72 (Kuder-Richardson formula 20) for the score on their feelings and 0.73 (Cronbach’s α) for the severity of feelings.

The second section assessed the presence of 20 different stressors, using a four-point Likert-type scale (0=very minimal; 1= slight; 2=moderate; 3=very much). The internal consistency coefficients were 0.93 (Kuder-Richardson formula 20) for the number of stressors and 0.93 (Cronbach’s α) for the stress severity.

The third section assessed the perceived effectiveness of a set of 14 direct and indirect factors in reducing the participant’s level of stress during the crisis period. The perceived effectiveness was rated using a four-point Likert-type scale as follows: 0=not at all effective; 1=mildly effective; 2=moderately effective; 3=extremely effective. The internal consistency coefficient (Cronbach’s α) for the effectiveness score was 0.85.

Section four explored the eventual active coping strategies deployed by the participants. It consisted of 13 items, rating the frequency at which the given strategy was used by the participant, from 0=never to 3=always. The internal consistency coefficients were 0.78 (Kuder-Richardson Formula 20) for the number of stressors and 0.80 (Cronbach’s α) for the rating of coping strategies.

The fifth section of 10 hypothetical incentives and evaluated their hypothetical impact on promoting the nurse’s willingness to participate in any future COVID-19 or other epidemic. The answers were rated on a four-point scale (0=not at all important to 4=most important). The internal consistency coefficients were 0.93 (Kuder-Richardson Formula 20) for the number of motivational factors and 0.93 (Cronbach’s α) for the rating of motivational factors.

The questionnaire items were adapted by the researchers to fit the context of COVID-19 and were supplemented with demographic and professional data, including age, gender, nationality, living arrangement, work experience, and so on. The final version underwent face and content validity checks by the researchers and two other independent healthcare professionals.

Procedure

All nurses in the institution received an invitation via email to participate in the study. This email included a link to the questionnaire, which had been adapted for online completion through Google Forms. Participation was entirely voluntary, and participants were informed of their right to withdraw at any point while completing the questionnaire. The nurses submitted their responses anonymously, and only completed questionnaires were included in the study's analysis.

Ethical considerations

Informed consent was obtained from all participants, ensuring that their participation was voluntary and that they understood the study objectives and procedures. Confidentiality and anonymity of participants were ensured throughout the study procedure. Data storage was secure, and access was limited to the research team. The study received ethical approval from the local ethics committee (#409-20).

Statistical analysis

The data were analyzed using Statistical Packages for Social Sciences (SPSS) version 24 (IBM Corp., Armonk, NY). Descriptive statistics were carried out and results are presented as means and standard deviations (SD) of the items' scores. Additionally, agreement rates for each item were calculated, defined as the frequency and percentage of responses indicating levels 1-3, which represent any degree of agreement from the participants with the given item.

## Results

Participants’ characteristics

Three hundred and fifteen nurses participated in this study. The ages of the participants varied, with the majority (54.3%) being in the 31-40 years age category, a minority (5.4%) were in the younger age group, while the remaining participants belonged to the older age group. Majority of the nurses were females (88.9%), non-Saudi (93.7%), and living alone (63.5%). The majority of the participants had 6-15 years of work experience (62.5%). Inpatient wards (31.4%) and emergency department (22.9%) were the most frequent departments (Table 1).

**Table 1:** Socio-demographic characteristics of nurses (N=315)

Study Data	N (%)
Age group	
21 – 30 years	17 (05.4)
31 – 40 years	171 (54.3)
41 – 50 years	75 (23.8)
51 – 60 years	52 (16.5)
Gender	
Male	35 (11.1)
Female	280 (88.9)
Marital status	
Single	89 (28.3)
Married	222 (70.5)
Divorced	04 (01.3)
Nationality	
Saudi	20 (06.3)
Non-Saudi	295 (93.7)
Work experience	
0 – 5 years	68 (21.6)
6 – 15 years	197 (62.5)
>15 years	50 (15.9)
Living arrangement	
Alone	200 (63.5)
Living with spouse	31 (09.8)
Living with family	84 (26.7)
Primary location of work	
Inpatient ward	99 (31.4)
Outpatient clinic	41 (13.0)
Emergency department	72 (22.9)
Isolation wards	45 (14.3)
Critical care unit	55 (17.5)
Other	03 (01.0)

Patterns of emotions during COVID-19 crisis

The most commonly experienced emotions were feeling of responsibility and ethical duty (97.5%), nervousness and fear (83.8%), anger toward the increasing workload compared to staffs not involved in COVID-19 care (73.3%), and the feeling of being avoided by staff not involved in COVID-19 care (70.2%). This was associated with discontent about the overtime working hours among 69.5%. Consequently, 84.4% of the participating nurses reported curtailing their contact with the COVID-19 patients. On the other hand, 86.7% were expecting a financial compensation for their duty during the outbreak and 66.6% appreciated the compensation. The mean (SD) scores of the items along with the agreement rates are presented in Table 2.

**Table 2:** Pattern of emotions experienced by the nurses during COVID-19 pandemic (N=315)

Emotion	Score, mean (SD)	Agreement rate, N (%)
You felt that you had to do your job as it was your professional and ethical duty	2.63 (0.71)	307 (97.5)
You felt nervous and scared during COVID-19 pandemic	1.84 (1.06)	264 (83.8)
You appreciated financial compensation after the pandemic	1.29 (1.11)	208 (66.0)
You were unhappy to do overtime	1.48 (1.20)	219 (69.5)
You appreciated special recognition for your job by the hospital	1.39 (1.12)	214 (67.9)
You expected financial compensation during the outbreak	2.12 (1.07)	273 (86.7)
You tried curtailing your contact with the COVID-19 (e.g. shorten your trips to patients' room)	1.77 (0.99)	266 (84.4)
You thought of quitting your job	1.24 (1.20)	185 (58.7)
You felt the employees not directly exposed to COVID-19 avoided you?	1.41 (1.10)	221 (70.2)
You noticed that employees outside your unit were avoiding COVID-19	1.62 (1.23)	222 (70.5)
If optional, would you have chosen to work in a unit where you would not be exposed to COVID-19	1.51 (1.27)	203 (64.4)
You would quit your job if COVID-19 outbreak recurred	0.69 (0.91)	135 (42.9)
You felt angry that your workload increased when compared to employees not exposed to COVID-19	1.43 (1.07)	231 (73.3)
You thought of calling in sick	1.12 (1.05)	187 (59.4)
You called in sick at least once	0.92 (1.03)	160 (50.8)

Stressors among nurses during COVID-19

Although all the explored stressors were highly reported, the most frequently reported ones were related to the nurses’ own safety, or the safety of their families and colleagues. These include seeing their own colleagues being infected and intubated (95.2%), lack of adequate protective measures (93%), the possibility of transmitting the infection to relatives (93%), and the eventual consequences of a small mistake or lapse in concentration (92.4%). The other cluster of important stressors was related to the perceived uncontrollability of the pandemic, such as the shortage of staff (95.2%), knowing the pandemic was still uncontrolled (94%), and watching news of new cases in the media (94%). The mean (SD) scores of the items along with the agreement rates are presented in Table 3.

**Table 3:** Stressors among nurses during COVID-19 pandemic (N=315)

Factors causing stress	Score, mean (SD)	Agreement rate, N (%)
Seeing your colleagues getting intubated	2.52 (0.78)	300 (95.2)
You could transmit COVID-19 to your family or friends	2.38 (0.91)	293 (93.0)
Small mistake or lapse in concentration could infect you or others	2.35 (0.95)	291 (92.4)
Taking care of your own colleagues who are sick from COVID-19	1.98 (1.07)	267 (84.8)
Seeing patients with Covid-19 die in front of you	2.38 (0.98)	286 (90.8)
Not knowing when the Covid-19 pandemic will be under control	2.27 (0.92)	296 (94.0)
Every time you were exposed to a new COVID-19 patient	2.04 (1.07)	271 (86.0)
Lack of treatment for COVID-19	2.18 (0.95)	293 (93.0)
Seeing news of new cases of COVID-19 reported in TV/newspaper	2.17 (0.93)	296 (94.0)
You were emotionally exhausted	2.1 (0.97)	286 (90.8)
You had physical stress/fatigue	1.98 (1.03)	271 (86.0)
Seeing your colleagues displaying COVID-19 like symptoms	2.07 (0.96)	290 (92.1)
You developed respiratory symptoms and feared that you had COVID-19	1.6 (1.19)	234 (74.3)
You could get COVID-19 infection from a patient in the hospital	2.15 (1.00)	288 (91.4)
Conflict between your duty and your own safety	2.1 (1.02)	283 (89.8)
Seeing colleagues stressed or afraid	2.1 (0.91)	294 (93.3)
Getting screened for COVID-19 Infection after exposure	1.85 (1.09)	268 (85.1)
You felt that you were not having adequate protective measures (including enough negative pressure rooms)	2.17 (0.97)	293 (93.0)
You had to wear protective gear on a daily basis	2.11 (1.06)	276 (87.6)
Shortage of staff at times	2.35 (0.89)	300 (95.2)
Total stress score (mean ± SD)	38.5 ± 17.5	-

Factors that contributed to reducing nurses’ stress

The improvement of health status of infected colleagues (98.1%) or patients (97.5%) was the most common factor associated with the reduction in nurses’ stress. These were followed by factors related to the working environment including the presence of clear guidelines for infection prevention in the hospital (95.6%), confidence in the hospital staff in case of being infected (94.9%), and positive attitude from colleagues (94.3%). Furthermore, the decrease in media reported cases contributed in reducing the stress among 95.2% of the participants. The mean (SD) scores of the items along with the agreement rates are presented in Table 4.

**Table 4:** Factors that reduced nurses’ stress during COVID-19 pandemic (N=315)

Factors that helped to reduce stress	Score, mean (SD)	Agreement rate, N (%)
Positive attitude from colleagues in your department	2.27 (0.89)	297 (94.3)
None of the staff getting COVID-19 after starting strict protective measures	1.8 (0.95)	288 (91.4)
Improvement in patient’s condition	2.19 (0.72)	307 (97.5)
Colleagues who were infected getting better	2.49 (0.67)	309 (98.1)
Protective equipment provided to you by hospital	1.98 (0.88)	294 (93.3)
Clear guidelines from hospital for infection prevention	2.35 (0.82)	301 (95.6)
Your family members or friends outside the hospital did not get COVID-19	1.99 (1.09)	266 (64.4)
Decrease in COVID-19 cases reported in the news	2.17 (0.87)	300 (95.2)
Likelihood that you would get extra compensation for your exposure to COVID-19	1.75 (1.15)	243 (77.1)
All healthcare professionals working together on the frontline	2.35 (0.92)	292 (92.7)
Confidence in the hospital staff in case you got sick from COVID-19	2.17 (0.85)	299 (94.9)
Not have to do overtime	1.87 (1.03)	278 (88.3)
Sharing jokes or humour among colleagues	2.08 (1.01)	279 (88.6)
Getting free meals from the hospital in your unit	1.22 (1.30)	161 (51.1)

Personal coping strategies

Among the 13 enumerated coping strategies, five were almost systematically deployed by the nurses (>95% of the participants). These included reading about COVID-19 prevention and transmission, strict adherence with PPE, escape-avoidance activities, seeking family and friends support, and avoidance of public places to minimize the risk of exposure. The other next frequent (90 - 95%) coping strategies including considering every patient as a COVID-19 case, compliance with other hygiene rules, and self-motivation toward keeping a positive attitude. The mean (SD) scores of the items along with the agreement rates are presented in Table 5.

**Table 5:** Personal coping strategies used by the nurses to alleviate stress (N=315)

Coping strategies used by the nurses	Score, mean (SD)	Agreement rate, N (%)
Followed strict personal protective measures (e.g. mask, gown, hand washing etc.)	2.72 (0.63)	309 (98.1)
Kept separate clothes for work/use disposable scrubs provided by Hospital to minimize transmission	2.42 (0.94)	292 (92.7)
Considered every patient admitted to the hospital as having COVID-19	2.27 (0.92)	296 (94.0)
Read about Covid-19 prevention and mechanism of transmission	2.62 (0.67)	311 (98.7)
Avoided going out in public places to minimize exposure from COVID-19	2.57 (0.78)	303 (96.2)
Did relaxation activities, e.g. involved in prayers, sports, exercise etc.	2.26 (0.97)	283 (89.8)
Chatted with family and friends to relieve stress and obtain support	2.62 (0.75)	303 (96.2)
Taking to yourself and motivate her to face the COVID-19 pandemic with a positive attitude	2.29 (0.93)	289 (91.7)
Got help from family physicians or other doctors to reduce your stress and get reassurance	1.55 (1.17)	224 (71.1)
Tried to be busy at home in activities that would keep your mind away from COVID-19	2.19 (0.84)	305 (96.8)
Avoided doing overtime to reduce exposure to Covid-19patients in hospital	1.45 (1.14)	223 (70.8)
Avoid media news about Covid-19and related fatalities	1.30 (1.11)	208 (66.0)
Vented your emotions by crying, screaming etc.	1.40 (1.11)	222 (70.5)

Motivational factors for future pandemics

Cure or vaccine availability for the disease was the most frequent determinant for the nurses’ commitment in future pandemics, claimed by 93.3% of the participants. The other frequent determinants included family support (91.4%), supply of adequate PPE by the hospital (90.8%), and exemption from overtime (90.2%). The mean (SD) scores of the items along with the agreement rates are presented in Table 6.

**Table 6:** Motivational factors to encourage the continuation of work in future pandemics (N=315)

Motivational factors for future pandemic	Score, mean (SD)	Agreement rate, N (%)
Similar adequate personal protective equipment supplied by the Hospital	2.36 (0.98)	286 (90.8)
Available cure or vaccine for the disease	2.52 (0.85)	294 (93.3)
Family support	2.61 (0.88)	288 (91.4)
Compensation to the family if disease-related death at work	2.38 (1.05)	274 (87.0)
Financial recognition of efforts	2.34 (1.08)	272 (86.3)
Disability benefits if disabled from the disease	2.26 (1.10)	271 (86.0)
Recognition from management and supervisors for the extra efforts	2.3 (1.04)	280 (88.9)
Psychiatric help and therapy are made available in the workplace to help reduce stress and anxiety	2.15 (1.10)	267 (84.8)
No need to do overtime	2.27 (1.03)	284 (90.2)
Reduced working hours during outbreaks	2.23 (1.08)	274 (87.0)

## Discussion

Nurses’ emotions during COVID-19 pandemic

The current study explored the common emotions and stressors reported by nurses during the first wave of the COVID-19 crisis and determined the key strategies they employed to preserve their motivation and mental well-being in the work setting. The nursing staff is probably the category of HCWs that is most directly exposed to patients, both in times of crisis and under normal circumstances. There is substantial evidence showing that HCWs who are in direct contact with patients in case of epidemics undergo significant stress and suffer from short- and long-term emotional distress [32-34]. Although Khalid et al. argued that each infectious disease is unique and has its own features, evidence shows that healthcare providers will be exposed to emotional stress whenever they become in direct contact with patients during outbreaks such as SARS and MERS [25]. This indicates that frontline nurses are at high risk of work-related burnout regardless of the type of outbreak.

The present showed that the nurses’ emotions during COVID-19 were dominated by a sense of duty and ethical responsibility toward their care mission. Similarly, Khalid et al. demonstrated that innate professional and ethical obligation was a highly common sentiment in HCWs, found in 80% of them, during the MERS-CoV epidemic [25]. Furthermore, this was the main reason why none of the nurses had resigned from our institution since the majority decided to continue working during the pandemic because of their ethical and professional commitment to their community. Such moral principles are very important for HCWs in the context of crisis, to prevent thoughts of quitting the job. A meta-analysis study found that approximately one-third of nurses who worked during the COVID-19 pandemic had intentions to leave their positions [35].

Regarding negative emotions, nervousness, and fear were remarkably common, concurring concords with findings from other studies that explored the emotional experience lived by HCWs during pandemics and outbreaks [25,36]. It is expected that the nurses reported fear feelings, as during outbreaks, healthcare staff are worried about themselves as well as about their relatives, colleagues, and patients from being infected [36]. Consequently, these worries caused a shortening in the contact time with patients as observed during the MERS epidemic [25], which was noted to be one of the coping strategies adopted by nurses in the present study.

Another commonly reported negative emotion is the feeling of being avoided by colleagues who were not directly exposed to COVID-19 patients. The first wave of COVID-19 was associated with a rising stigma toward patients and HCWs, including nurses. Such a phenomenon was transcultural, and nursing staff from different regions of the world have suffered stigmatization, both in their workplace and social environment. This further impacted the psychological well-being of nurses, causing social isolation impacting their self-image, and impeded mental health seeking in some cases [37,38]. On the other hand, the impression of being avoided by our nurses was probably exaggerated by social distancing measures imposed within the care institution to limit the intrahospital transmission of COVID-19.

Consistent with other studies, this study demonstrated that the nursing staff in the studied institution, although worked with the same nurse-to-patient ratio (1:4) as before the pandemic, felt a greater workload when dealing with COVID-19 patients compared to nurses who were not exposed to those patients [25,39,40]. This perceived extra workload may be related to a stricter implementation of the preventive precautions before, during, and after dealing with infected patients. Perception of increased workload can significantly enhance anxiety among workers, compromising the overall experience of healthcare provision [40]. Most of our participants expected additional financial compensation for continuing their duty during COVID-19. If not met, these expectations may exacerbate the feeling of injustice and impact the commitment among nurses. A study from Qatar showed that benefiting from financial compensation was positively associated with the level of nurses’ motivation to provide care for COVID-19 patients [41].

Stressors and stress-relieving factors during COVID-19 pandemic

Findings from this study showed that the main stressors faced by nurses during COVID-19 were connected to personal safety and the safety of family, colleagues, and patients. Watching colleagues and patients getting infected or decreasing from infection was the source of immense anxiety among nurses. In the context of the first COVID-19 wave, this was associated with the lack of knowledge about the disease and unfamiliarity with the changing, often conflicting information. The epidemiological emergency imposed by the pandemic was prone to a lot of confusion and contradictory recommendations, which was perceived as a major stressor by HCWs and other first responders [13,42]. Consistently, studies conducted during the SARS and MERS epidemics revealed safety concerns to be a principal cause of stress and pressure among HCWs [25,36]. Such feelings of confusion and fear are associated with a feeling of insufficiency towards the nurse’s own role, which exposes them to a high risk of burnout [39]. These stressors were fed by the perceived uncontrollability of the disease, as demonstrated in our findings, where staff shortage and the increasing number of new cases shown in the media were highly prevalent stressors reported by more than 94% of the nurses.

Regarding stress-relieving factors, the most commonly reported was the recovery of infected colleagues and improvement in hospitalized patients’ condition. Witnessing the improvement of COVID-19-infected colleagues and patients generates optimistic emotions about the curability of the disease, as shown in previous data [36]. Further stress-reducing factors were the availability of proper prevention guidelines, strong feelings of trust among the staff, preservation of positive attitudes within the hospital, and the relative decrease in COVID-19 cases. These factors highlight the importance of a secure and positive work environment in reducing psychological distress among nurses, as reported in other studies [25,27,36]. Ensuring a strong and effective communication strategy and providing updated and clear guidelines are crucial to minimize stress during global health crises such as the COVID-19 pandemic, and to improve the resilience of the care system [43].

Personal coping and motivation strategies

Nurses used a range of strategies to maintain their resilience towards the COVID-19 crisis situation and combat the related stress. The most commonly used coping strategies included increasing their knowledge regarding COVID-19 prevention and transmission and applying strict preventive measures, both in hospitals and community. These observations indicate that a conscious and proactive focus on self-protection from COVID-19 is the most common and effective coping strategy among the nursing staff. Several other coping mechanisms are reported in the literature and shown to positively impact nurses’ and HCWs’ resilience during health crises [25,30,44,45].

Additional mechanisms such as considering all patients to be positive for COVID-19, complaining about irresponsible attitudes from others, and trying to motivate themselves were also frequently reported. Coping strategies involving support from relatives can raise the feelings of security, love, and motivation, and reduce the social isolation imposed by stigma [46].

Regarding sources of motivation for working in future pandemics, the availability of vaccines or potential treatments was the most cited source by the participants. Besides, adequate family support and PPE supply were also perceived to be important motivators for the participants. It is important to identify factors that reinforce work motivation among HCWs and alleviate their hesitation to prevent staff shortages in times of health crises. The present study and other reports demonstrated that motivators related to staff safety and disease severity are the major determinants of the willingness to accept work or intention to leave work during the health crisis [47,48]. Another important observation is the prominent need for protection and mental and social support to maintain the level of engagement and the sense of duty among nurses.

Implications for practice, policy, and research

The study underscores the pivotal role of nurses, especially their innate sense of duty and ethical responsibility, during healthcare crises such as the COVID-19 pandemic. To address the emotional challenges they face, it is imperative to enhance emotional and psychological support mechanisms. This includes both training that emphasizes the ethical dimensions of their roles and measures to counteract workplace stigmatization. Healthcare institutions must ensure that nurses working directly with infected patients are not marginalized, through comprehensive education about risks and protective measures. Furthermore, acknowledging the heightened workload and stress they undergo, suitable financial compensations and benefits should be extended, and effective safety protocols with the consistent provision of PPE should be a priority.

The need for effective communication shows to be another key implication. The study highlights feelings of confusion and fear stemming from conflicting or rapidly changing information. Addressing this issue requires transparent, consistent, and evidence-based communication about disease progress and protective measures. By promoting a positive workplace atmosphere that emphasizes recoveries and provides updates on infected colleagues, optimism can be instilled. Furthermore, continuous training and education can serve as vital coping mechanisms, with an emphasis on increasing knowledge about prevalent diseases and their prevention.

Looking toward future pandemic preparedness, institutions and healthcare decision-makers should consider enhancing HCWs' commitment considering the potential risk of hesitation. Assuring rapid development and availability of treatments, uninterrupted PPE supply chains, and solid psychological and social support systems can alleviate these hesitations. Offering these resources ensures that nurses remain engaged and motivated and maintain their sense of duty amidst health crises.

Regarding research implications, several perspectives can be explored. Comparative analyses across different infectious disease outbreaks like SARS, MERS, and COVID-19 can pinpoint consistent stressors and coping strategies. Longitudinal studies could track the long-term emotional impacts on nurses post-pandemic, and the depth of stigmatization they experience warrants deeper exploration. The efficacy of coping mechanisms, the role of institutional support, the determination of optimal workloads during crises, and global perspectives on nurses' challenges are also crucial areas for future research. Such a comprehensive approach can provide valuable insights for informed decision-making and robust institutional support during health crises.

Limitations

The present study has a few significant limitations. Using a descriptive approach, the study only assessed the prevalence of each emotion, stressor, and coping strategy, which does not enable assessing the impact and significance of each parameter. Although beyond the study’s scope, assessing the levels of stress would have enabled a better assessment of the significance of the stressors and stress-relieving factors. The second limitation, which is inherent to the exclusive quantitative approach, is the use of restricted lists for emotions, stressors, and coping strategies, which limited the scope of the answer. The third limitation is related to information bias, which is the risk of overestimating negative feelings and perceptions, as the majority of participants were immigrants with a short professional experience and living alone, which may further impact the participants’ self-efficacy and mental and social well-being during the COVID-19 crisis. On the other hand, the workforce belonged to diverse ethnic backgrounds, which can be considered a strength of the study.

## Conclusions

The first wave of COVID-19 exerted tremendous psychological stress on nurses, generating a set of negative feelings mainly related to safety concerns, uncertainties about the disease, and profound stigma and social isolation. Positive attitudes and work commitment were maintained by the nurses’ sense of duty towards their respective roles, as well as by a positive working environment, notably the presence of clear guidelines, adequate PPE, and solidarity among HCWs. Besides, the contribution of proactive coping appears to be fundamental, involving a set of cognitive and behavioral mechanisms, most of them being directly related to own safety. The COVID-19 crisis constitutes a valuable teaching experience to strengthen crisis plans, by giving high priority to safety assurance and psychological preparedness and wellbeing among the staff, especially the first-line responders such as nurses.
